# Transcriptome differences between two sister desert poplar species under salt stress

**DOI:** 10.1186/1471-2164-15-337

**Published:** 2014-05-04

**Authors:** Jian Zhang, Jianju Feng, Jing Lu, Yongzhi Yang, Xu Zhang, Dongshi Wan, Jianquan Liu

**Affiliations:** State Key Laboratory of Grassland and Agro-Ecosystems, School of Life Sciences, Lanzhou University, Lanzhou, 730000 Gansu China; Xinjiang Production & Construction Corps Key Laboratory of Protection and Utilization of Biological Resources in Tarim Basin, College of Plant Science, Tarim University, Alar 843300 Xinjiang, China

**Keywords:** *P. euphratica*, *P. pruinosa*, Salt tolerance, Salinity stress, Transcriptome, Differentially expressed genes, Alternative splicing

## Abstract

**Background:**

*Populus euphratica* Oliv and *P. pruinosa* Schrenk (Salicaceae) both grow in dry desert areas with high summer temperatures. However, *P. euphratica* is distributed in dry deserts with deep underground water whereas *P. pruinosa* occurs in deserts in which there is underground water close to the surface. We therefore hypothesized that these two sister species may have evolved divergent regulatory and metabolic pathways during their interaction with different salt habitats and other stresses. To test this hypothesis, we compared transcriptomes from callus exposed to 24 h of salt stress and control callus samples from both species and identified differentially expressed genes (DEGs) and alternative splicing (AS) events that had occurred under salt stress.

**Results:**

A total of 36,144 transcripts were identified and 1430 genes were found to be differentially expressed in at least one species in response to salt stress. Of these DEGs, 884 and 860 were identified in *P. euphratica* and *P. pruinosa*, respectively, while 314 DEGs were common to both species. On the basis of parametric analysis of gene set enrichment, GO enrichment in *P. euphratica* was found to be significantly different from that in *P. pruinosa*. Numerous genes involved in hormone biosynthesis, transporters and transcription factors showed clear differences between the two species in response to salt stress. We also identified 26,560 AS events which were mapped to 8380 poplar genomic loci from four libraries. GO enrichments for genes undergoing AS events in *P. euphratica* differed significantly from those in *P. pruinosa*.

**Conclusions:**

A number of salt-responsive genes in both *P. euphratica* and *P. pruinosa* were identified and candidate genes with potential roles in the salinity adaptation were proposed. Transcriptome comparisons of two sister desert poplar species under salt stress suggest that these two species may have developed different genetic pathways in order to adapt to different desert salt habitats. The DEGs that were found to be common to both species under salt stress may be especially important for future genetic improvement of cultivated poplars or other crops through transgenic approaches in order to increase tolerance of saline soil conditions.

**Electronic supplementary material:**

The online version of this article (doi:10.1186/1471-2164-15-337) contains supplementary material, which is available to authorized users.

## Background

Salinity and drought stresses are the two most important environmental factors limiting plant growth and development in semiarid and arid areas [1]. Over 100 countries in the world have been identified as being affected by salinity [2], and the scale of the problem seems to be increasing at an alarming rate [3]. Salinity, together with drought, has far-reaching implications for food security, economic sustainability and the irreplaceable biodiversity of any affected area, and it is anticipated that these challenges will be exacerbated by the projected impact of climate change. The effects of water-insufficiency stresses have been studied extensively; they limit water and micronutrient uptake and lead to closure of stomata, decline in carbon metabolism, stunted growth, ion/salt toxicity and reduced yield [3, 4].

For plants to survive under such conditions, they must sense and respond to these abiotic stresses rapidly and in a complex manner [5], through signalling and regulatory pathways [3, 4, 6] mediated by abscisic acid [7] or ethylene [8], generally resulting in altered expression of transcription factors [9], and in many cases in increased expression of genes encoding products required for osmoregulation, cell protection and/or acclimation [10–15]. These modifications may lead to changes in signal transduction, ionic homeostasis, scavenging of reactive oxygen species, accumulation of compatible solutes and growth regulation [3, 6, 16–18]. A common strategy for the identification of overall changes in gene expression under salt stress is to compare the transcriptomes of the targeted species or cultivars using microarrays and/or RNA-Seq technologies [19]. A plethora of comparisons between salt-sensitive and salt-tolerant cultivars of model and non-model plant species, including *Arabidopsis*[20–22], rice [23], poplar [24–27], tomato [28], potato [29], *Medicago truncatula*[30], sugarcane [31] and olive [32], have been reported to date. These studies have identified more than 30 families of transcription factors and numerous enzyme-encoding genes involved in responses to salt stress [33, 34]. However, overall changes in gene expression and physiological responses to salt stress vary greatly between different species, particularly between sensitive and non-sensitive pairs of related species [35–39]. It is often difficult to ascertain whether these differences were caused by divergence during the course of evolution or were brought about through adaptive differentiation. It is therefore of interest to compare the overall changes in gene expression that occur in sister species under salt stress, as this will minimise phylogenetic effects.

Here we examine differences in the transcriptomes of two sister desert poplar species under salt stress. *Populus* serves as a model for elucidating physiological and molecular mechanisms of stress tolerance in tree species [40–42]. Both *P. euphratica* and *P. pruinosa* grow in dry deserts with high summer temperatures [43–46]. Both species can tolerate high salinity and survive NaCl concentrations of more than 300 mM [47] in nutrient solution, and *P. euphratica* has been used as a model species for studying abiotic responses to salt or drought stress [27, 48–50]. In addition to differences in leaf and hair morphology between the two species, they also occur in different types of habitat. *P. euphratica* is found in dry deserts with deep underground water while *P. pruinosa* is distributed in deserts where the underground water is closer to the surface, and therefore more accessible, but also saltier near ancient or extant rivers. It is likely that these two species have diverged due to ecological differentiation, in spite of ongoing gene flow [46].

In order to test whether regulatory and metabolic pathways in these two species have diverged during their adaptive interactions with salt and other stresses, the transcriptomes of callus subjected to 24 h of salt stress, and control callus samples, from *P. euphratica* and *P. pruinosa* were compared in order to identify differentially expressed genes (DEGs) and alternative splicing (AS) events that occurred in response to salt stress. Our results revealed that these two poplar species have both common and species-specific patterns of gene expression under salt stress. The dynamic transcriptome expression profiles of these sister species under salt stress obtained in this study may provide useful insights to inform further analyses of the mechanism of high salinity tolerance in plants. In addition, the genes found to be differentially expressed under salt stress in both species may facilitate the identification of key genes as potentially suitable targets for biotechnological manipulation with the aim of improving poplar salt tolerance.

## Results and discussion

### Analysis and mapping of Illumina-Solexa sequencing tags

**Figure 1:**
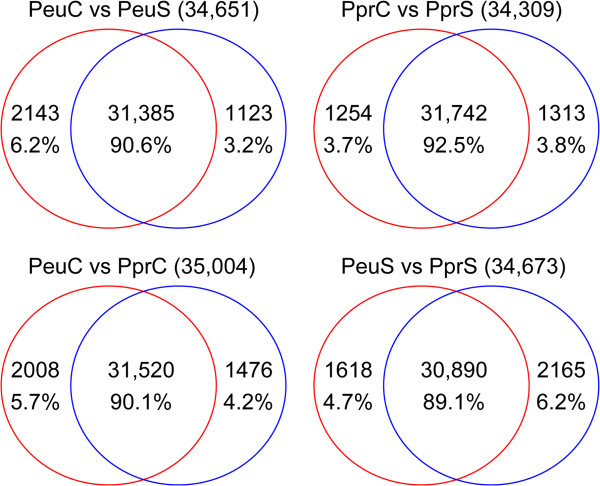
**Venn diagrams showing mapped genes expressed in each possible pair out of the four libraries.** PeuC, *P. euphratica* control callus; PeuS, *P. euphratica* salt-stressed callus; PprC, *P. pruinosa* control callus; PprS, *P. pruinosa* salt-stressed callus.

We used the Illumina-Solexa sequencing platform to sequence the *P. euphratica*[27] and *P. pruinosa*[51] transcriptomes obtained from the four treatments, including two unstressed callus samples as controls (*P. euphratica* control callus, PeuC; *P. pruinosa* control callus, PprC) and two salt-stressed callus samples as treatments (*P. euphratica* salt-stressed callus, PeuS; *P. pruinosa* salt-stressed callus, PprS). After removing low-quality sequences and trimming adapter sequences, ~28 million 75-bp paired-end clean reads were generated from each of the cDNA libraries in the Illumina Genome Analyzer runs (Table 1). These tags from the four digital gene expression (DGE) libraries were mapped to the available *P. trichocarpa* transcript sequences. Approximately 80% of the tags had matches. Most (79.2–82.4%) of the tags with matches were unique tags (matching only one poplar locus), while the remainder (~17.6–20.8%) were non-unique (matching more than one poplar locus) or unaligned. For more detailed investigation of gene expression in the different treatments, only unique tags were used in the analysis. In total, 36,144 transcripts were identified from the four conditions. The transcripts identified accounted for 80.3% of the 45,033 annotated genes in poplar. In both control and salt stress treatments, the numbers of mapped genes in *P. euphratica* (33,528 and 32,508 genes) were found to be similar to those in *P. pruinosa* (32,996 and 33,055 genes, respectively) (Table 1). We further compared the mapped genes among the four treatments (PeuC, PeuS, PprC and PprS), and found that ~89.1% of them were present in at least two treatments (Figure 1).

**Table 1:** **Summary of the Illumina-Solexa sequencing tags and their matches in the** ***P. trichocarpa*** **genome**

Samples	Matched genes (%)	Aligned tags		Unaligned tags (%)	Total clean tags
		Unique (%)	Non-unique (%)		
PeuC	33,528 (74.5)	22,704,962 (82.4)	229,952 (0.8)	4,621,235 (16.8)	27,556,149
PeuS	32,508 (72.2)	23,375,397 (80.6)	297,806 (1.0)	5,314,269 (18.4)	28,987,472
PprC	32,996 (73.3)	22,298,805 (79.2)	241,822 (0.9)	5,599,162 (19.9)	28,139,789
PprS	33,055 (73.4)	23,027,167 (81.0)	364,836 (1.3)	5,046,367 (17.7)	28,438,370

PeuC, *P. euphratica* control callus; PeuS, *P. euphratica* salt-stressed callus; PprC, *P. pruinosa* control callus; PprS, *P. pruinosa* salt-stressed callus.

### DEGs in the two species under salt stress

**Figure 2:**
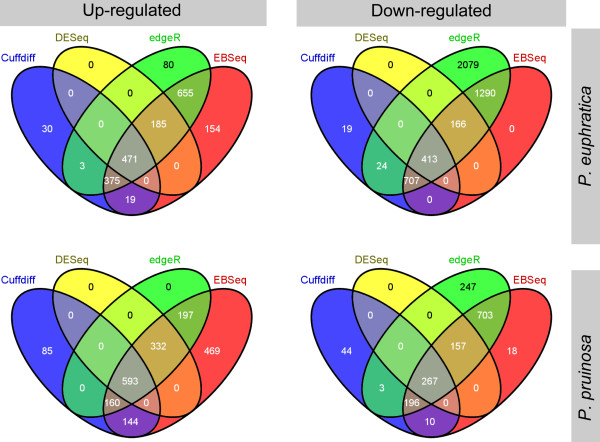
**Comparison of four metrics for classifying DEGs.** Venn diagrams of the numbers of up-regulated (left) and down-regulated (right) genes identified by four comparisons of control callus and salt-stressed callus from *P. euphratica* (top) and *P. pruinosa* (bottom).

To identify global transcriptional changes occurring under salt stress, we applied four independent metrics to identify genes that were differentially expressed between the 24-h salt-stressed callus and control callus samples in *P. euphratica* and *P. pruinosa*. For each metric, we selected those DEGs whose expression profiles met three criteria: (i) the FPKM value was ≥1 in either of the libraries, (ii) log_2_ (FPKM_salt_/FPKM_control_) was > 1 or < -1, and (iii) the adjusted *p*-value (FDR) was < 0.05. In this study, DEGs with higher expression levels in salt-stressed callus when compared with control callus samples were termed ‘up-regulated’, while those with lower expression levels in salt-stressed callus were termed ‘down-regulated’. There were 471 and 593 genes identified by all metrics as being up-regulated in *P. euphratica* and *P. pruinosa*, respectively, and 413 and 267 genes identified by all metrics as down-regulated in *P. euphratica* and *P. pruinosa*, respectively (Figure 2). There were more up-regulated DEGs in *P. pruinosa* than in *P. euphratica*, while there were more down-regulated DEGs in *P. euphratica* than in *P. pruinosa*.

**Figure 3:**
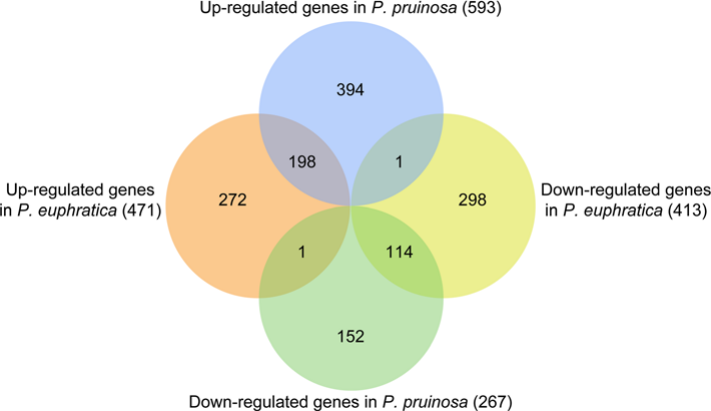
**Number of DEGs in** ***P. euphratica*** **and** ***P. pruinosa*** **.** The numbers of DEGs that were exclusively up- or down-regulated in one species are shown in each circle. The numbers of DEGs with the same or opposite pattern of expression changes between the two species are shown in the overlapping regions. The total numbers of up- or down-regulated genes in each species are shown outside the circles.

The DEGs identified were classified into eight clusters according to their expression patterns (Figure 3, Additional file 1). Of these eight clusters, four were up-regulated or down-regulated exclusively in a single species, as follows: up-regulated exclusively in *P. euphratica* (272 DEGs) or in *P. pruinosa* (394); down-regulated exclusively in *P. euphratica* (298) or in *P. pruinosa* (152). The remaining four clusters consisted of genes that were up- or down-regulated in the two species; two of these clusters showed similar co-regulation patterns whereas the other two showed opposing regulation patterns. In the two clusters with similar co-regulation patterns, 198 DEGs were co-up-regulated and 114 DEGs were co-down-regulated in the two species. Within the co-up-regulated clusters, only one transcript (POPTR_0013s12880.1) was undetectable in the calli of the two species under unstressed conditions (Additional file 1), suggesting that this gene is expressed specifically under salt stress in both species. In the two clusters with opposing patterns of regulation, only 1 DEG was up-regulated in *P. euphratica* but down-regulated in *P. pruinosa*, and only 1 DEG was down-regulated in *P. euphratica* but up-regulated in *P. pruinosa*. This result suggested that our integrated DEG identification was sensitive and reliable.

### Confirmation of differentially expressed candidate genes by qRT-PCR analysis

**Figure 4:**
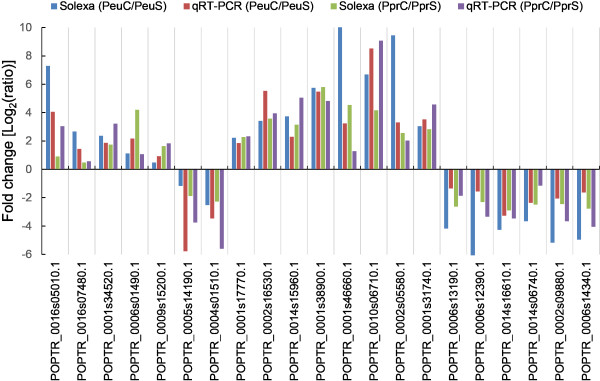
**Expression pattern validation of selected genes by qRT-PCR.** Expression changes of 21 DEGs in the salt-stressed calli relative to the control calli were measured by qRT-PCR. The transcriptional level of candidate genes was examined by real time PCR with three biological replications and *actin* was used as an internal control. Results were present as target/reference ratios normalized by the calibrator. No significant differences were shown between qRT-PCR and the Illumina data (Pearson’s correlation coefficient *r* = 0.8). The *Y*-axis indicates the fold change of transcript abundance in salt-stressed callus relative to the control callus. PeuC, *P. euphratica* control calli; PeuS, *P. euphratica* salt-stressed calli; PprC, *P. pruinosa* control calli; PprS, *P. pruinosa* salt-stressed calli.

To confirm the gene expression inferred from RNA-seq, a total of 21 candidate DEGs with salt-related process were selected for the qRT-PCR analyses, comprising 7 DEGs exclusively regulated in a single species, 8 co-up-regulated and 6 co-down-regulated in the two species (Figure 4). Although the exact change did not exactly match each other, the expression trends of all 21 genes from qRT-PCR and Illumina-Solexa RNA sequencing analyses were largely consistent (Pearson’s correlation coefficient *r* = 0.8), demonstrating the reliability of the RNA-seq results (Figure 4).

### Gene functional categories of two species under salt stress

**Figure 5:**
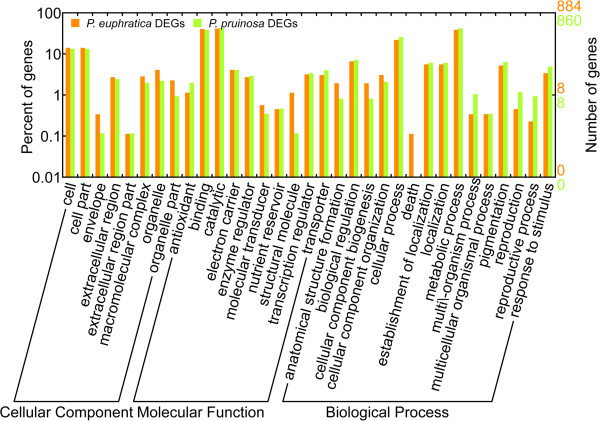
**Gene ontology (GO) annotation of salt-responsive genes compared between** ***P. euphratica*** **and** ***P. pruinosa.*** WEGO was used to produce the graph. We divided the sets into the three major GO domains: biological process, cellular component and molecular function, and the number (right y-axis) and percentage (left y-axis) of genes were calculated.

Firstly, an overview of the main results was obtained by WEGO and the DEGs were assigned to GO terms in the three component ontologies (Figure 5). Then, groups of genes with functions involved in salt responses were identified using parametric analysis of gene set enrichment (PAGE) (Table 2). GO enrichment in *P. euphratica* was significantly different from that in *P. pruinosa*. In the Cellular Component ontology, ‘apoplast’ (GO:0044464) appeared to respond to salt stress in both species; while ‘cell part’ (GO:0044464) and ‘cell’ (GO:0005623) were enriched only in *P. euphratica*; whereas ‘extracellular region’ (GO:0005576), ‘external encapsulating structure’ (GO:0030312) and ‘cell wall’ (GO:0005618) were enriched only in *P. pruinosa*. In the Molecular Function ontology, ‘cofactor binding’ (GO:0048037), ‘coenzyme binding’ (GO:0050662), ‘peptidase inhibitor activity’ (GO:0030414) and ‘endopeptidase inhibitor activity’ (GO:0004866) were enriched in both species, while another nine terms from the Molecular Function ontology were enriched exclusively in *P. euphratica*. Six terms from the Biological Processes ontology were enriched exclusively in *P. euphratica* and three terms were enriched in both species. The GO terms enriched in *P. euphratica* were related to responses to stress and metabolic processes, and the most highly enriched term was ‘response to stress’ (GO:0006950). We also used singular enrichment analysis (SEA) to identify functional groups of genes differentially expressed in the two species under salinity (Additional file 2). GO enrichment for genes up-regulated or down-regulated exclusively in *P. euphratica* was significantly different from that in *P. pruinosa*. The detected differences suggested that these two desert poplars might have developed different genetic pathways for adaptation to differentiated salty desert habitats.

**Table 2:** **Gene ontology (GO) enrichment analyses for salt-responsive genes compared between** ***P. euphratica*** **and** ***P. pruinosa*** ^**1**^

GO term	Onto^2^	Description	Number	***P. euphratica***			***P. pruinosa***		
				Z-score	Mean^3^	FDR	Z-score	Mean	FDR
GO:0006950	P	Response to stress	50	7.2	5.1	4.50e-11	3.1	1.8	0.21
GO:0050896	P	Response to stimulus	54	6.7	4.6	1.60e-09	3.1	1.8	0.21
GO:0006952	P	Defense response	16	6.4	7.7	1.50e-08	3.2	2.9	0.11
GO:0009607	P	Response to biotic stimulus	15	5.9	7.4	4.20e-07	3.4	3.1	0.063
GO:0009308	P	Amine metabolic process	22	3.5	3.7	0.047	2.3	2	1
GO:0044264	P	Cellular polysaccharide metabolic process	13	-3.6	-4.3	0.036	-4.6	-3	0.00034
GO:0044042	P	Glucan metabolic process	13	-3.6	-4.3	0.036	-4.6	-3	0.00034
GO:0006073	P	Cellular glucan metabolic process	13	-3.6	-4.3	0.036	-4.6	-3	0.00034
GO:0044260	P	Cellular macromolecule metabolic process	159	-3.6	-1	0.035	-2.2	0.11	1
GO:0048037	F	Cofactor binding	67	8.1	4.9	3.90e-14	3.8	1.9	0.011
GO:0050662	F	Coenzyme binding	53	7.9	5.4	2.00e-13	3.5	2	0.034
GO:0030414	F	Peptidase inhibitor activity	21	7.9	8.3	2.50e-13	4.3	3.2	0.0015
GO:0004866	F	Endopeptidase inhibitor activity	21	7.9	8.3	2.50e-13	4.3	3.2	0.0015
GO:0050660	F	FAD binding	33	7.4	6.3	1.40e-11	2.9	2	0.37
GO:0016491	F	Oxidoreductase activity	191	6.6	2.5	2.60e-09	2.3	1.1	1
GO:0004857	F	Enzyme inhibitor activity	32	6.5	5.7	6.50e-09	3.4	2.3	0.066
GO:0030234	F	Enzyme regulator activity	33	6.2	5.4	3.80e-08	3.2	2.1	0.13
GO:0030246	F	Carbohydrate binding	24	4.5	4.6	0.00052	2.3	1.9	1
GO:0004497	F	Monooxygenase activity	42	4	3.2	0.0052	1.1	1.1	1
GO:0003824	F	Catalytic activity	504	3.7	1	0.019	0.93	0.72	1
GO:0046906	F	Tetrapyrrole binding	64	3.5	2.3	0.042	0.12	0.65	1
GO:0020037	F	Heme binding	64	3.5	2.3	0.042	0.12	0.65	1
GO:0017171	F	Serine hydrolase activity	13	-3.4	-4.1	0.059	-3.5	-2.1	0.036
GO:0008236	F	Serine-type peptidase activity	13	-3.4	-4.1	0.059	-3.5	-2.1	0.036
GO:0016758	F	Transferase activity, transferring hexosyl groups	16	-3.1	-3.3	0.2	-3.8	-2.1	0.011
GO:0016762	F	Xyloglucan:xyloglucosyl transferase activity	10	-3.1	-4.2	0.2	-4.2	-3.1	0.0023
GO:0048046	C	Apoplast	10	-3.1	-4.2	0.047	-4.2	-3.1	0.00054
GO:0044464	C	Cell part	169	-3.2	-0.87	0.03	-1.1	0.36	1
GO:0005623	C	Cell	169	-3.2	-0.87	0.03	-1.1	0.36	1
GO:0005576	C	Extracellular region	12	-2.4	-3	0.33	-3.3	-2.1	0.02
GO:0030312	C	External encapsulating structure	18	-2.8	-2.8	0.11	-3.8	-1.9	0.0033
GO:0005618	C	Cell wall	18	-2.8	-2.8	0.11	-3.8	-1.9	0.0033

^1^The 1430 DEGs were analyzed by parametric analysis of gene set enrichment (PAGE).

^2^Onto, Gene Ontology domain; P, biological process; F, molecular function; C, cellular component.

^3^Mean, mean log_2_ expression ratio, >0 represents up-regulation, <0 represents down-regulation.

### Differences in expression of hormone-related genes in the two species under salt stress

**Figure 6:**
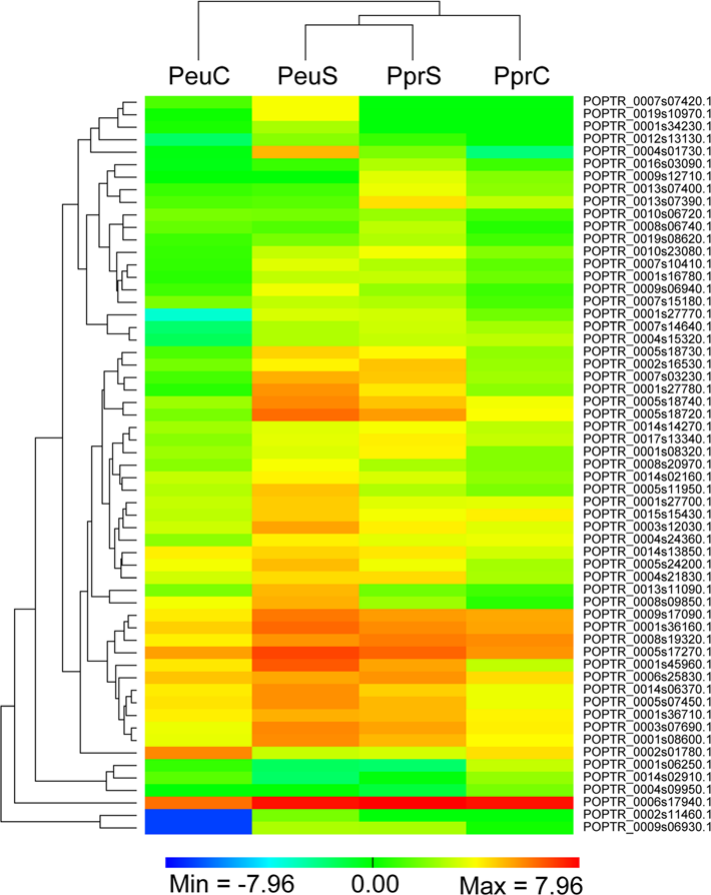
**Hierarchical clustering of 58 genes related to plant hormone biosynthesis.** Hierarchical cluster analysis was conducted using the software PermutMatrix v1.9.3 and displayed as a log-(FPKM) value for the intensity of expression of each DEG. FPKM, number of fragments per kilobase of exon per million fragments mapped; PeuC, *P. euphratica* control callus; PeuS, *P. euphratica* salt-stressed callus; PprC, *P. pruinosa* control callus; PprS, *P. pruinosa* salt-stressed callus.

Using the Kyoto Encyclopedia of Genes and Genomes (KEGG) database as our source of annotations, 583 out of 803 *Populus* genes annotated as being involved in hormone biosynthesis [52] were detected in the four libraries and 59 of these genes were differentially expressed in either *P. euphratica* or *P. pruinosa* under salt stress (Additional file 3). Among these hormone biosynthesis-related DEGs, 37 were identified in *P. euphratica*, of which 36 were up-regulated and one was down-regulated during salt stress, while 39 were identified in *P. pruinosa*, including 36 up-regulated and 3 down-regulated DEGs; only 17 were co-regulated in the two species. Hierarchical clustering of the 59 hormone-related DEGs showed overall differences between *P. euphratica* and *P. pruinosa* in response to salt stress (Figure 6). Under salt stress, most hormone-related DEGs were co-up-regulated or co-down-regulated in both species. Interestingly, three ABA metabolism-related genes (*ABA1*, POPTR_0007s10980.1; and two genes encoding 9-cis-epoxycarotenoid dioxygenase (*NCEDs*), POPTR_0011s11370.1 and POPTR_0001s40420.1) were up-regulated exclusively in *P. pruinosa*.

### Differences in expression of transporter-encoding genes in the two species under salt stress

On the basis of annotations in the database of *Arabidopsis thaliana* transporter proteins (http://www.membranetransport.org/all_type_btab.php?oOID=atha1), a total of 99 genes differentially regulated according to all metrics in either *P. euphratica* or *P. pruinosa* during salt stress were categorized as transporters (Additional file 4). Among these, 49 DEGs were identified in *P. euphratica*, of which 25 were up-regulated and 24 down-regulated during salt stress, while 66 were differentially regulated in *P. pruinosa*, consisting of 51 up-regulated and 14 down-regulated DEGs, and 16 were co-regulated in the two species. For example, we found that POPTR_0003s13470.1, POPTR_0008s14670.1 and POPTR_0006s11590.1, which are homologous to *Arabidopsis thaliana* Na^+^/H^+^ antiporter 18 (AT5G41610, CHX18), potassium transporter 6 (AT1G70300, KUP6) and ABC transporter (AT1G66950, ABCG39), respectively, were co-up-regulated in both species. However, POPTR_0005s04660.1 and POPTR_0014s12700.1, which are homologous to *Arabidopsis thaliana* sodium/hydrogen exchanger 2 (AT3G05030, NHX2) and potassium transporter 5 (AT4G13420, HAK5) genes, were up-regulated only in *P. euphratica*. In contrast, POPTR_0004s23680.1 and POPTR_0013s08110.1, which are homologous to *Arabidopsis thaliana* chloride channel protein CLC-c (AT5G49890, CLC-C) and potassium transporter 2 (AT2G40540, KT2) genes respectively, were up-regulated exclusively in *P. pruinosa*. These results corroborate previous findings [27, 53–55], and confirm that genes encoding proteins such as sodium and potassium ion transmembrane transporters, and chloride channel and ABC transporters, which are important for maintaining and re-establishing homeostasis in the cytoplasm, are induced to high levels in response to salinity stress [16].

### Differences in expression of transcription factor genes in the two species under salt stress

We identified 4016 transcription factors in *Populus trichocarpa* and classified them into 92 families (Additional file 5) based on published annotations. A total of 115 genes that were differentially regulated in either *P. euphratica* or *P. pruinosa* during salt stress were categorized as transcription factors (Additional file 6). Of these, 59 DEGs were identified in *P. euphratica*, including 24 that were up-regulated and 35 down-regulated during salt stress, while 73 DEGs were identified in *P. pruinosa*, of which 52 were up-regulated and 21 down-regulated. Only 17 DEGs were co-regulated in both species (Table 3, Additional file 6). Several of the transcription factors, such as AP2/ERF and bZIP, which are known to be induced by stress in model plant species (*Arabidopsis thaliana* and rice) [56, 57], were highly expressed in response to salinity stress in *P. euphratica* or *P. pruinosa*.

**Table 3:** **Transcription factors differentially expressed in the two species under salinity stress**

Transcription factor family	***P. euphratica***	***P. pruinosa***
AP2/ERF	13	11
bZIP	1	2
MYB	3	9
WRKY	5	8
NAC	7	8
C2H2	2	2
bHLH	3	0
MYB-like	1	2
GARP-G2-like	1	0
HB, Homeobox	2	2
GRAS	2	2
Other	19	27
Total	59	73

### The co-up-regulated DEGs in the two species under salt stress and allele mining

A total of 198 co-up-regulated DEGs in the two species were identified in salt stress (Additional file 1) and the important ones were selected and listed in Table 4. The candidate genes identified in the present study contained both the previously reported salt-responsive genes and some species-specific ones. Of them, most genes were involved in and highly enriched in functional categories such as response to stress, signal transduction, transmembrane transport, transcriptional regulation and basic metabolic processes (Additional files 1 and 2). These findings are beneficial to allele mining of two poplar species related to their common or differentiated response to stressed habitats in the future. Allele mining based on the candidate genes were found to be important in dissecting naturally selected allelic variations that controlled differentiated traits [58, 59]. In addition, promoters are found to play a key role in gene regulation, and any change in these regions will change gene expression and the controlled traits. Therefore, the identified variations through such an approach may be mainly located in the promoter regions [60]. Overall, the co-up-regulated DEGs identified in the present study provide critical genetic bases for further allele mining, functional analyses and transgenic practices for developing the salt-tolerant poplars and crops.

**Table 4:** **List of co-up-regulated DEGs in the two species under salinity stress**

Transcript name	***Arabidopsis***	Gene annotation	FPKM				log2 Ratio(PeuS/PeuC)	log2 Ratio(PprS/PprC)
			PeuC	PeuS	PprC	PprS		
POPTR_0008s17940.1	AT3G04120	glyceraldehyde-3-phosphate dehydrogenase	17.64	430.78	19.83	321.82	4.61	4.02
POPTR_0006s25280.1	AT5G25880	malate dehydrogenase (NADP+)	48.70	190.54	28.89	85.17	1.97	1.56
POPTR_0007s14250.1	AT4G37870	phosphoenolpyruvate carboxykinase [ATP]	82.37	565.15	92.96	253.92	2.78	1.45
POPTR_0006s11590.1	AT1G66950	ABC transporter G family member 39	3.02	36.26	5.99	17.88	3.59	1.58
POPTR_0001s26210.1	AT4G16260	catalytic/cation binding/hydrolase	0.05	132.37	0.00	15.30	11.41	13.90
POPTR_0015s05290.1	AT1G73480	alpha/beta-hydrolase domain-containing protein	1.90	17.84	0.63	10.20	3.23	4.02
POPTR_0016s01570.1	AT3G21760	UDP-glycosyltransferase-like protein	2.87	20.08	2.06	13.49	2.81	2.71
POPTR_0006s12220.1	AT3G53150	UDP-glucosyl transferase 73D1	1.27	20.29	1.56	18.09	4.00	3.54
POPTR_0005s21690.1	AT2G33710	ethylene-responsive transcription factor ERF112	0.93	27.49	1.18	23.49	4.89	4.32
POPTR_0002s04020.1	AT3G23240	ethylene-responsive transcription factor 1B	1.44	28.29	3.29	14.91	4.29	2.18
POPTR_0008s14670.1	AT1G70300	Potassium transporter 6	35.29	191.46	55.07	133.58	2.44	1.28
POPTR_0010s09370.1	AT3G22740	homocysteine S-methyltransferase 3	0.13	16.64	0.31	12.10	7.04	5.28
POPTR_0015s11130.1	AT5G13080	putative WRKY transcription factor 75	24.38	143.60	30.56	286.38	2.56	3.23
POPTR_0010s10010.1	AT5G26340	sugar transport protein 13	5.48	116.42	8.20	72.52	4.41	3.15
POPTR_0001s09000.1	AT4G11650	osmotin-like protein OSM34	0.07	245.80	1.04	165.57	11.83	7.32
POPTR_0004s22170.1	AT5G60700	glycosyltransferase family protein 2	0.09	4.58	0.19	3.96	5.68	4.40
POPTR_0009s15100.1	AT1G08250	arogenate dehydratase 6	37.86	158.76	31.10	154.12	2.07	2.31
POPTR_0017s04590.1	AT5G53970	tyrosine aminotransferase	29.34	253.35	24.46	201.65	3.11	3.04
POPTR_0006s16610.1	AT4G28940	Phosphorylase-like protein protein	6.75	153.46	4.72	113.79	4.51	4.59

### A comparison of DEGs identified by our results and other transcriptome studies of the salt-stressed poplars

In order to test the consistency of DEGs across different treatments and approaches, we compared DEGs between our results and other available transcriptome studies of the salt stressed poplars. Ottow et al. [48] examined changes in transcript levels of various genes known to be involved in salt or general stress signaling or adaptation in *P. euphratica* leaves by dot-blot expression. They identified nine genes with significant changes in response to salt stress. Some of them were be confirmed in the present study, for example, galactinol synthase 2 (GolS2, POPTR_0013s00730.1), calcineurin B-like protein 4 (CBL 4, POPTR_0015s01550.1), alternative oxidase 1A (POPTR_0012s01630.1) and 1-aminocyclopropane-1-carboxylate oxidase (POPTR_0011s00970.1) (Additional file 1). Galactinol synthase (GolS) catalyzes the first step in the biosynthetic pathway of raffinose oligosaccharides using galactose and myo-inositol as substrates and this gene was also up-regulated in plants under cold, heat, drought, and salt stress [21, 61, 62]. Significant increases in galactinol synthase and alternative oxidase after salt stress point to shifts in carbohydrate metabolism and suppression of reactive oxygen species in mitochondria under salt stress [48]. In addition, Gu et al. [63] identified 54 genes with altered transcript accumulation in the salt-stressed *P. euphratica* by microarray hybridization. The genes of them, responsible for hydroxyproline-rich glycoprotein, carbonic anhydrase 2, cytochrome P450, aquaporin, sucrose synthase and aspartate aminotransferase were confirmed in these present study. These genes were also revealed to be salt-responsive in other studies [26, 27, 64].

The drought responses of plants are similar to those in response to salinity because both stresses lead to physiological water deficit [65]. Bogeat-Triboulot et al. [66] provided a comprehensive analysis of *P. euphratica* subjected to gradual soil water depletion, and observed 110 regulatory and protective genes involved in long-term response to drought. Similar results were also found by Cohen et al. [25] and Tang et al. [67]. Among them, those genes involved in metabolites of proline, raffinose, galactose, inositol and sucrose under drought stress were found to have changed their expressions in response to salt stress in the presnet study. An increase in galactinol, raffinose and stachyose content may have improved osmoprotection and ROS scavenging when poplars were stressed by drought or salt. However, in the present study, we identified numerous more transcripts with significant up-regulations in both poplars when stressed, including UDP-glycosyltransferase-like protein, FAD-binding and BBE domain-containing protein, putative nucleoredoxin 1, and glyceraldehyde-3-phosphate dehydrogenase. All these newly identified genes should have also played an important role during salt adaptation of two species. Their functions and molecular mechanisms need further clarifications in the future.

### Alternative splicing of transcripts in the two species under salt stress

**Figure 7:**
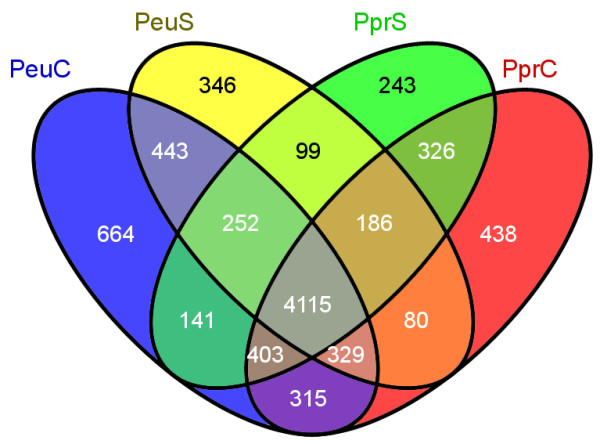
**Number of loci showing AS events in** ***P. euphratica*** **and** ***P. pruinosa*** **.** The numbers of loci undergoing AS events in each species and treatment are shown. PeuC, *P. euphratica* control callus; PeuS, *P. euphratica* salt-stressed callus; PprC, *P. pruinosa* control callus; PprS, *P. pruinosa* salt-stressed callus.

Finally, to investigate the role of alternative splicing (AS) in response to salt stress, we conducted a survey of transcript isoforms across the four libraries and examined six common types of ‘alternative splicing events’. For each of these event types, reads deriving from specific regions can be used to identify the expression of one alternative isoform or the other (Table 5). We identified 26,560 AS events which were mapped to the 8380 poplar genomic loci from the four libraries, suggesting that ~20% of 40,668 loci in poplar are potentially subject to AS. This observed AS percentage is comparable to the percentage of the genes shown to undergo AS in *A. thaliana* (21.8%) and rice (21.2%) [68]. In both control and salt stress treatments, the number of genes exhibiting AS events in *P. euphratica* (6662 and 5850 genes) is similar to that in *P. pruinosa* (6192 and 5765 genes), respectively. In addition to the AS loci (4115) common to both species, around 346 and 243 of the loci show AS events only in either *P. euphratica* or *P. pruinosa* in response to salt stress (Figure 7). We further classified those genes that underwent AS events on the basis of functional ontology. GO enrichment for the genes displaying AS events exclusively in *P. euphratica* was significantly different from that for genes undergoing AS in *P. pruinosa* (Additional file 7).

**Table 5:** **Alternative splicing events in response to salt stress in** ***P. euphratica*** **and** ***P. pruinosa***

Type of event	PeuC	PeuS	PprC	PprS	Total
Skipped exon	13,323	12,130	11,655	10,749	20,334
Retained intron	2867	2195	2182	1776	4795
Alternative 5’ splice site (A5SS)	274	294	282	269	529
Alternative 3’ splice site (A3SS)	250	232	251	230	430
Alternative first exon (AFE)	162	129	148	142	222
Alternative last exon (ALE)	157	145	154	144	250
Total AS events	17,033	15,125	14,672	13,310	26,560
Loci having AS events	6662	5850	6192	5765	8380

PeuC, *P. euphratica* control callus; PeuS, *P. euphratica* salt-stressed callus; PprC, *P. pruinosa* control callus; PprS, *P. pruinosa* salt-stressed callus.

## Conclusions

Our transcriptional profiling analysis revealed numerous genes that were differentially expressed in both *P. euphratica* and *P. pruinosa* under salt stress. The differential expressions of the selected genes inferred from RNA-seq were confirmed by qRT-PCR data. Gene ontology analyses of these DEGs suggested that GO enrichment in *P. euphratica* was significantly different from that in *P. pruinosa*. We found that numerous genes involved in hormone biosynthesis, or encoding transporters or transcription factors, showed different expression patterns between these two species under salt stress. These differences suggest that these two desert poplars may have developed species-specific pathways for adaptation to salinity during the course of ecological speciation in their different salty desert habitats. The results of our comparative analyses imply that different species, even sister species, may employ different genetic pathways to cope with salt stress. This suggests that it may be more difficult than previously anticipated to design salt-tolerant plant cultivars [69, 70]. In order to develop cultivars with high salt tolerance, particular attention should be paid to those genes that are differentially expressed in two or more different species under salt stress. Such genes can be used to facilitate genetic improvement of crops, including cultivated poplars, for growth on saline soils.

## Methods

### Gene expression data

Paired-end RNA-seq reads for control callus and salt-stressed callus of *P. euphratica* and *P. pruinosa*, which were obtained by Qiu et al. [27] and Zhang et al. [51], respectively, were downloaded from the NCBI sequence read archive (accession numbers SRX025571, SRX025568, SRX245887 and SRX245885).

We cultured *P. euphratica and P. pruinosa* calli induced from the shoot under the same conditions. We then replaced the growth medium for one set with the fresh medium and the same medium but supplemented with 100 mM NaCl (salt stress) for another set. We harvested both sets of calli 24 h later. The calli from *P. euphratica* and *P. pruinosa* had the same subculture generation and time and they were highly comparable in terms of physiological state. After RNA extraction and quality determination, we constructed the paired-end cDNA libraries with insert sizes of 200 base pair (bp), and then sequenced the cDNA using an Illumina (San Diego, CA, USA) Genome Analyzer platform according to the manufacturer’s protocols with a read length of 75 bp in two lanes. Image output data from the sequencer was transformed into raw sequence data by base calling.

Raw reads generated by Illumina Genome Analyzer were initially processed to obtain clean reads. We first cleaned raw sequence reads by removing exact duplicates from both sequencing directions. We further cleaned reads by removing adapter sequences as well as reads with too many (>8) unknown base calls (N), low complexity, and low-quality bases (>50% of the bases with a quality score ≤5). Cleaned reads from each library were used for later differential expression analysis in this study.

### Initial mapping of reads

To determine the level of gene expression, Bowtie2 [71] was used to align RNA-seq reads from the control and salt-stressed samples to transcript sequences from *Populus trichocarpa* Torr. & A. Gray [41], using annotation files downloaded from http://www.phytozome.net/poplar (JGI *Populus trichocarpa* v2.2). No more than a 1 bp mismatch was allowed when taking into account differences between the two species. Reads that mapped to reference sequences from multiple genes were filtered out. The remaining clean reads, which were considered to be distinct, were used for further analysis. Transcript abundances were calculated using eXpress [72], which outputs read counts and the number of fragments per kilobase of exon per million fragments mapped (FPKM) [73]. Transcripts with FPKM values < 1 in both libraries were filtered out and not subjected to further analysis.

### Identification of differentially expressed genes

To identify differentially expressed genes (DEGs) in control callus and salt-stressed callus from *P. euphratica* and *P. pruinosa*, we applied four independent, widely used tools: Cuffdiff [73], DESeq [74], edgeR [75], and EBSeq [76]. Cuffdiff takes a nonparametric, annotation-guided approach to estimating the means and variances of transcript FPKM values under different conditions, using Student’s *t*-tests to identify differentially expressed transcripts [73]. In contrast, DESeq, edgeR and EBSeq estimate the means and variances of raw read counts under a negative binomial distribution and use exact tests to identify differentially expressed transcripts. The main difference between DESeq, edgeR and EBSeq is that they use different statistical approaches to estimate variance [74–76]. After the *p*-values for each expressed genes were obtained by the four tools, the false discovery rate (FDR) was used to justify the *p*-value by the function *p.adjust* in R. Sequences were deemed to be differentially expressed if log_2_(FPKM_salt_/FPKM_control_) > 1 or < -1, and the adjusted *p*-value (FDR) was < 0.05 as identified by all four metrics.

### Functional annotation through BLAST2GO and KEGG

Gene Ontology (GO) terms were assigned to the identified genes by the blast2GO pipeline [77] using NCBI databases, followed by functional classification using the WEGO software package [78]. For the comparative analysis of DEGs between *P. euphratica* and *P. pruinosa* in response to salinity, singular enrichment analysis (SEA) and parametric analysis of gene set enrichment (PAGE) were performed using the agriGO program (http://bioinfo.cau.edu.cn/agriGO) [79] with the default parameters, using the *P. trichocarpa* gene models as background, followed by multiple testing with Bonferroni correction (corrected P-value < 0.05). PermutMatrix (Version 1.9.3; http://www.lirmm.fr/~caraux/PermutMatrix/index.html) was used to cluster genes related to plant hormone biosynthesis according to their mean normalized intensity values [80].

### Validation of DEG Expression with Quantitative Real-time PCR (qRT-PCR)

In order to validate the reliability of RNA-Seq experiments, a total of 21 candidate DEGs highly related to salt stress were selected for qRT-PCR test. These genes were chosen for the qRT-PCR analysis based on two criteria: (i) gene’s expression patterns between these two species under salt stress should be similar; (ii) it should have only one BLAST hit when searching against genes of *Arabidopsis thaliana* to exclude paralogs. A total of 0.5 μg of DNase I-treated total RNA was converted into single-stranded cDNA using a Prime-Script 1st Strand cDNA Synthesis Kit (TaKaRa, Dalian, China). The cDNA templates were then diluted 20-fold before use. The quantitative reaction was performed on a CFX96 Real-Time PCR Detection System (Bio-Rad, Singapore) using SYBR Premix Ex Taq™ (TaKaRa, Dalian, China). PCR amplification was performed under the following conditions: 30 s at 95°C, followed by 40 cycles of 95°C for 15 s, 60°C for 30 s and then 72°C for 20 s. All primers were designed using PRIMER3 software and were listed in Additional file 8. Three biological replicates based the calli cultured from different individuals with the same subculture and physiological state were performed in order to exclude sampling errors. The relative expression levels of the selected DEGs normalized to an internal reference gene *actin* was calculated using 2^-ΔΔCt^ method [81].

### Identification of alternative splicing

We prepared a database of all possible splice junctions between annotated exons in each selected gene and identified new possible junctions using TopHat [82]. We combined these two databases, removing any redundancy between them, and then extracted junction sequences of width 65 bases on each side from all the above junctions. To evaluate which of these junctions were validated by our Illumina reads, we aligned reads from each library separately against the junction sequences, allowing up to one mismatch (in a read of 75 bp). If at least two reads aligned to a splice junction, we considered it to be validated.

Six different types of alternative mRNA processing events were analysed [83]. We first considered skipped exons (SE), in which one or more exons are spliced out of the mature message, and retained introns (RI), in which one or more introns are included in the message. Also included were alternative 5’ splice site (A5SS) and alternative 3’ splice site (A3SS) events, which are particularly difficult to interrogate by microarray analysis because the variably included region is often quite small. Finally, alternative last exons (ALEs) in which alternative use of a pair of polyadenylation sites results in distinct terminal exons, and alternative first exons (AFEs), where alternative promoter use results in mRNA isoforms with distinct 5’ UTRs, were considered.

## Electronic supplementary material

Additional file 1: **Eight clusters of DEGs under salt stress.** (XLSX 272 KB)

Additional file 2: **GO enrichment analyses for the eight clusters of DEGs under salt stress.** GO terms, identified by SEA analysis as significant, for co-regulated and species-specific DEGs up- or down-regulated in *P. pruinosa* and *P. euphratica* under salinity stress. The categories ‘down-regulated in *P. pruinosa* and up-regulated in *P. euphratica*’ and ‘up-regulated in *P. pruinosa* and down-regulated in *P. euphratica*’ were excluded because only one gene fell into each of these categories. (XLSX 22 KB)

Additional file 3: **Genes involved in the biosynthesis of plant hormones.** (XLSX 25 KB)

Additional file 4: **Ninety-nine DEGs were categorized as transporters.** (XLSX 33 KB)

Additional file 5: **Transcription factors in** ***Populus trichocarpa*** **.** (XLSX 115 KB)

Additional file 6: **One hundred and fifteen DEGs were categorized as transcription factors.** Each transcription factor contains known DNA-binding domains defined in the Pfam database. (XLSX 30 KB)

Additional file 7: **GO enrichment analyses for the 346 and 243 loci showing AS events solely in either** ***P. euphratica*** **or** ***P. pruinosa*** **in response to salt stress.** (XLSX 14 KB)

Additional file 8: **Primer used for real-time quantitative RT-PCR in this study.** (XLSX 14 KB)

## Abbreviations

DGE: Digital gene expression
GO: Gene ontology
KEGG: Kyoto Encyclopedia of Genes and Genomes
FPKM: Number of fragments per kilobase of exon per million fragments mapped
DEGs: Differentially expressed genes
PAGE: Parametric analysis of gene set enrichment
SEA: Singular enrichment analysis
FDR: False discovery rate
qRT-PCR: Quantitative real-time polymerase chain reaction.
